# Tunable stereoselectivity in a wireless electrochemical microreactor using natural chiral ionic liquids

**DOI:** 10.1039/d5gc04756k

**Published:** 2025-10-30

**Authors:** Sara Grecchi, Andrea Mezzetta, Lorenzo Guazzelli, Serena Arnaboldi

**Affiliations:** a Dipartimento di Chimica, Università degli Studi di, Milano Via Golgi 19 Milano 20133 Italy sara.grecchi@unimi.it serena.arnaboldi@unimi.it; b Dipartimento di Farmacia, Università di di Pisa Via Bonanno 33 Pisa 56126 Italy

## Abstract

A single drop of a natural chiral ionic liquid (NCIL) serves as a wireless electrochemical microreactor for enantioselective synthesis. This system yields high selectivity (>90% ee) and demonstrates dynamic stereocontrol, where the enantiomeric excess is directly modulated by the external electric field. This catalyst-free strategy introduces a new paradigm for sustainable, tunable asymmetric reactions.

## Abstract 2

A green and sustainable enantioselective electrosynthesis strategy using a drop of naturally derived chiral ionic liquid, harnessed within a polypyrrole tube microreactor acting as an efficient wireless pump.

## Graphical abstract

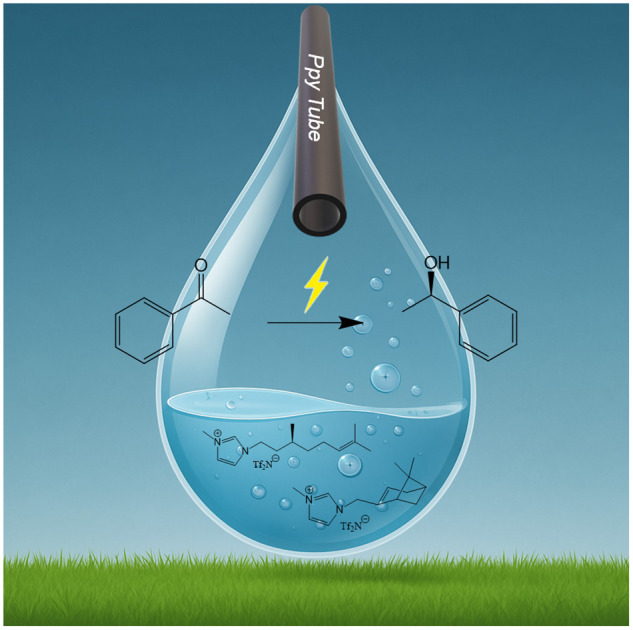

**Green foundation**

1. Our work advances green chemistry by introducing a catalyst-free platform for asymmetric synthesis using wireless electrochemistry. Employing renewable, terpene-derived ionic liquids as microreactors, this strategy avoids precious metal catalysts and hazardous reagents, replacing volatile organic solvents and operating under ambient conditions.

2. We achieved highly enantioselective (>90% ee) reduction of prochiral ketones with a theoretical atom economy of ∼100%. A key achievement is dynamic stereocontrol: the product's enantiomeric ratio is tuned in real-time by adjusting the electric field, thus replacing chemical modifiers with a physical parameter.

3. To become greener, the high Process Mass Intensity (PMI ≈ 783), dominated by workup solvents, must be reduced. Future research will target integrated, low-solvent purification and confirm the long-term recyclability of the NCIL and microreactor, enhancing process circularity and practical viability.

Ionic liquids (ILs) have emerged as versatile solvents and reaction media in various fields of chemistry due to their unique physicochemical properties, including low volatility, high thermal stability, and tunable polarity.^1,2^ Their ability to dissolve a wide range of organic and inorganic compounds, coupled with their non-flammability and negligible vapor pressure, makes them highly attractive for applications in sustainable chemistry.^3^ Among the various classes of ILs, chiral ionic liquids (CILs) have gained significant interest for their potential role in asymmetric synthesis, chiral separations, and enantioselective catalysis and electroanalysis.^4–8^

While synthetic CILs have been extensively studied, the development of natural chiral ionic liquids (NCILs) is an emerging and promising research area. NCILs, which are often derived from biomolecules such as amino acids, alkaloids, and terpenes, offer distinct advantages in terms of sustainability, biocompatibility, and reduced toxicity compared to their synthetic counterparts.^9,10^ The intrinsic chirality of these natural building blocks can be exploited to design novel NCILs with specific stereochemical properties, making them particularly useful for enantioselective processes in organic synthesis.^11^

In such field, NCILs serve as both solvents and co-catalysts in a variety of reactions, including asymmetric transformations, green oxidation–reduction processes, and transition-metal-catalyzed reactions.^12^ Their ability to influence reaction selectivity and efficiency has positioned them as valuable tools in modern synthetic methodologies. In addition to their role in traditional organic synthesis, NCILs have gained attention in electrosynthesis, where they facilitate electron transfer, stabilize reactive intermediates, and enable the development of environmentally safer electrochemical methodologies.^13,14^ Their ionic nature, combined with their ability to modulate reaction environments, makes them ideal candidates for electrochemical applications such as electrocatalysis, electroanalysis, and energy storage systems.^15,16^ In the field of electrochemistry, NCILs have demonstrated promising results as electrolytes and electrode modifiers, enhancing the stability, conductivity, and efficiency of various electrochemical devices. Their structured solvation environments and ability to form specific ion pairs contribute to improved charge transport properties, which are critical for applications in batteries, supercapacitors, and biosensors.^14–16^ Furthermore, their biocompatibility makes them particularly suitable for applications in biomedical electrochemistry, including biosensing and drug delivery devices.^17,18^ Enantioselective synthesis using electrochemical techniques offers several advantages over traditional methods, including reduced waste, lower costs, and milder reaction conditions. The ability to produce molecules with high enantiomeric purity is crucial in various fields, especially in pharmaceuticals, where enantiomers of a chiral active pharmaceutical ingredient can exhibit different biological activities.

This project explores a novel approach to enantioselective electrosynthesis, specifically the reduction of a prochiral precursor (acetophenone, AP) to a single enantiomer of a chiral compound (*R*- or *S*-phenylethanol, PE) using wireless bipolar electrochemistry (BE).^19^ This method combines the pumping effect of a miniaturized polypyrrole (Ppy) tube (synthetized as reported in Fig. S1), used as bipolar electrode (BPE)^20,21^ and activated by an electric field (*ε*), with the enantioselective capabilities of NCILs, used as chiral solvents/reaction media.

The NCILs used in this work are derived from citronellol and myrtenol, two natural and biodegradable terpenes commonly found in essential oils and are considered of low toxicity (the chemical structures are reported in Fig. 1). Their use aligns with the principles of green chemistry by replacing synthetic ILs with bio-based, safer alternatives. These NCILs facilitate enantioselective reduction due to favorable diastereomeric interactions within the microreactor. By applying a constant *ε*, enantioselective reduction occurs within the tube, and the products are collected and analyzed using chiral HPLC to determine enantiomeric purity and apparent product yield. This approach offers a promising alternative to traditional methodologies, minimizing toxic waste and reaction time while utilizing environmentally friendly solvents.

**Fig. 1:**
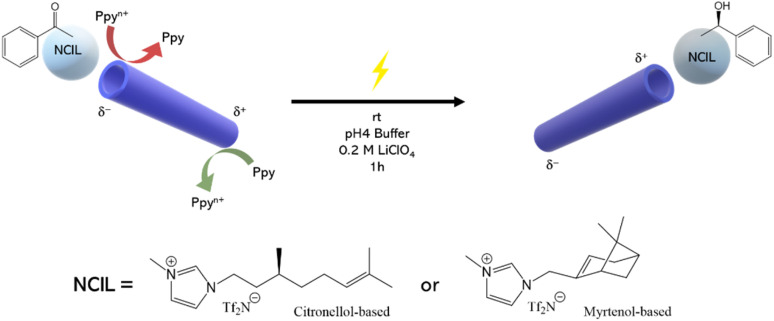
Schematic representation of the wireless asymmetric electroreduction of AP to PE *via* the enantioselective drop microreactor, along with the corresponding operating conditions and the reactions involved on the Ppy tube for the pumping mechanism. On the bottom the chemical structures of the two NCILs based on citronellol or myrtenol are also reported.

## Electrochemical characterization and chiral recognition

Preliminary electrochemical investigations using cyclic voltammetry (CV) were crucial for establishing the fundamental electrochemical behavior of the two NCILs and their potential utility in enantioselective electrochemistry. The observation of a reduction peak at approximately −2.8 V *vs.* Fc^+^|Fc for both the citronellol- and myrtenol-based NCILs is indicative of the reduction of the imidazolium cation (Fig. S2), a common structural feature in many ionic liquids. The determination of the electrochemical window is essential for defining the potential limits within which electrochemical reactions can be carried out without solvent interferences. The broad potential windows observed, extending to approximately 2 V in oxidation and −2 to −2.5 V (*vs.* Ag|Ag^+^) in reduction, highlight the electrochemical stability of these NCILs. This wide window is a significant advantage, as it allows for a greater range of electrochemical reactions to be performed, without involving side reactions of the chiral medium (Fig. S3).

To evaluate the enantioselective capabilities of the NCILs, CV measurements were conducted with *R*- and *S*-PE. The significant peak potential separation (Δ*E*_p_) observed between the *R*- and *S*-enantiomers is a direct measure of the NCIL's ability to differentiate between the two enantiomers (Fig. S4). The Δ*E*_p_ values of ∼400 mV for the citronellol-based NCIL and ∼470 mV for the myrtenol-based one indicate a substantial degree of chiral recognition. Furthermore, the favorable interaction indicated for *R*-PE suggests a preferential interaction between the *R*-enantiomer and the chiral environment provided by the NCILs in both cases. These results are consistent with the hypothesis that diastereomeric interactions between the chiral medium and the enantiomers lead to differences in their electrochemical behavior. These preliminary CV studies not only confirmed the enantioselective capabilities of the NCILs but also provided critical information for selecting appropriate potential values for the subsequent BE experiments aimed at enantioselective electrosynthesis.

## Wireless enantioselective reduction of acetophenone

The concept of wireless electromechanical pumping has emerged as a powerful tool for generating controlled unidirectional liquid flow.^20,21^ This mechanism leverages the reversible electromechanical properties of Ppy, along with its electrical tunability and processability. When a sufficient electric field is applied, the opposing charged ends of a hollow Ppy tube-shaped undergo shrinking and swelling.^20–22^ This asymmetrical modification of the tube's inner diameter induces a unidirectional flow based on the Bernoulli principle.

The use of a wireless, miniaturized microreactor enables significant reduction in reaction volume and reagent consumption (only a few μL of solvent), making this approach particularly attractive for applications requiring high efficiency with minimal environmental footprint.

Interestingly, in this case, it was not necessary to invert the applied electric field to prolong the interaction time (Video S1).

Additionally, the presence of charged species in the ionic liquid influenced its response to the applied electric field, leading to a more controlled and less immediate movement of the fluid. Unlike neutral solvents, where flow is primarily pressure-driven, the conduction in ionic liquids occurs mainly through ion migration, further reducing the overall transport rate. As a result, the prochiral compound remained in contact with the Ppy tube long enough for efficient enantioselective conversion, yielding a high enantiomeric excess (ee) without requiring adjustments to the electric field direction. This finding highlights the inherent advantages of chiral ionic liquids in wireless enantioselective electromechanical systems, offering new perspectives for their application in asymmetric synthesis and chiral separations.

**Fig. 2:**
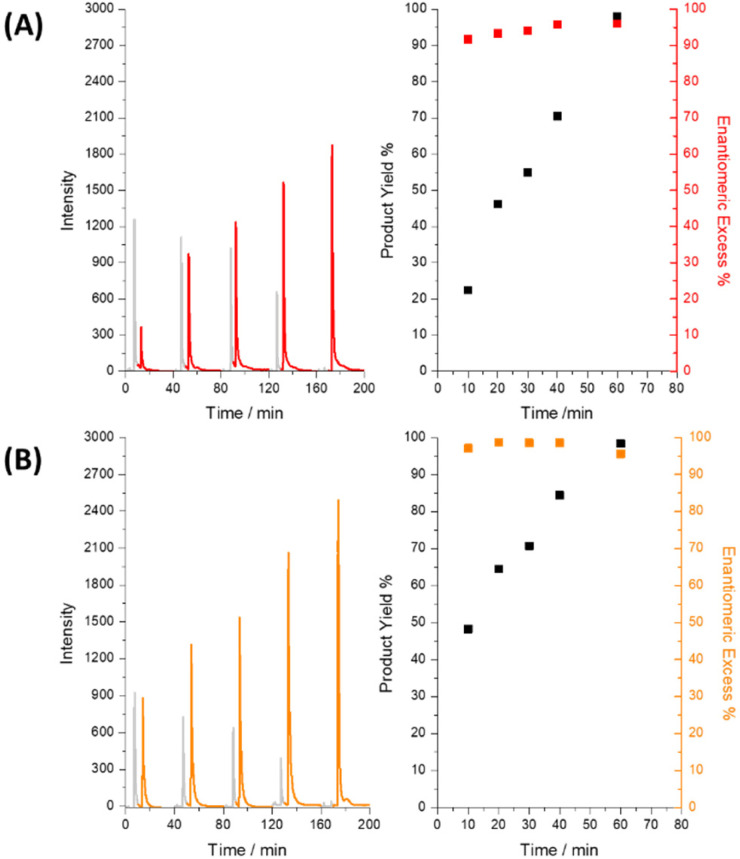
Chromatograms of the fractions collected at different reaction times (10, 20, 30, 40 and 60 minutes) from the *δ*^+^ extremity of the Ppy tube with citronellol- (A) and myrtenol-based (B) NCILs by applying a constant electric field of 2 V cm^−1^. The red and orange colors stand for *R*-PE, while the gray peaks are related to AP. On the right column, the corresponding enantiomeric excess (in red or orange) and apparent product yield (in black) as a function of the time of electrolysis.

The BE experiment, using a citronellol-based NCIL as the reaction medium, successfully demonstrated the enantioselective reduction of AP to *R*-PE (Fig. 2A). The Ppy tubes, synthetized with a procedure described in the SI, were characterized through SEM and elemental analysis. SEM images revealed an average outer diameter of ∼3 mm for the tubes, allowing for a higher injection volume, and a wall thickness of approximately ∼0.9 mm (Fig. S5). Probably due to the procedure, the inner tube surface appears rough as a result of the silver paint sacrificial layer, while the outer surface was more homogeneous. EDX analysis of Ppy tubes showed carbon and nitrogen as the main components, with trace silver (<1%) likely from residual silver paint (Fig. S6). Therefore, optimizing synthesis and washing to reduce silver residues could improve long-term stability and performance.

The HPLC analysis of the collected fractions provided direct evidence for the progress and selectivity of the electrochemical reduction. The chromatographic peaks were assigned to the corresponding enantiomers by comparing the results with those obtained from the pristine compounds (Fig. S7). The progressive decrease in AP concentration and the corresponding increase in *R*-PE concentration over time clearly illustrate the effective and selective reduction of AP to the desired *R*-enantiomer (Fig. 2) with high enantiomeric excess (ee) and conversion efficiency (>90%). Compared to traditional metal-based enantioselective hydrogenation or enzymatic resolution, this electrochemical approach eliminates the need for toxic or expensive catalysts, while maintaining excellent selectivity and conversion. This contributes to safer, greener synthetic protocols, particularly relevant for pharmaceutical intermediates. This observation is consistent with the preliminary CV results, which indicated a preferential interaction between the citronellol-based NCIL and the *R*-enantiomer of PE. The detection of residual AP within the tube following the experiment, through the analysis of the heptane washing, suggests that the reaction may not have proceeded to completion within the timeframe of the experiment (Fig. S8). This incomplete conversion could be attributed to several factors, including the viscosity of the NCILs, which may limit mass transport within the microreactor, or the applied electric field and reaction time. Further optimization of these parameters could potentially improve the conversion efficiency. Preliminary tests indicate that both the NCILs and the Ppy microreactor can be reused with minimal loss of activity. This observation is consistent with the known electrochemical stability of Ppy when operated within its reversible redox window, avoiding irreversible degradation mechanisms such as over-oxidation (SI, Section S6, for a detailed discussion on polymer stability and lifetime). This suggests potential for further optimization toward material recovery and circular use of components. However, the experiment demonstrates the feasibility of using BE in conjunction with NCILs for the enantioselective synthesis of chiral molecules. The BE measure was repeated using a myrtenol-based NCIL to further investigate the effect of the chiral selector on the enantioselectivity and efficiency of the reaction (Fig. 2B). Similar to the previous experiment, HPLC analysis confirmed the reduction of AP to *R*-PE. However, the myrtenol-based NCIL appeared to facilitate a more efficient and rapid conversion, as evidenced by the faster decrease in AP concentration and the more rapid increase in *R*-PE concentration (Fig. 2B). This observation aligns with the higher peak potential separation (Δ*E*_p_) observed in the enantioselectivity CV studies, suggesting that the myrtenol-based NCIL exhibits a stronger chiral discrimination and/or promotes a faster reaction rate.

The detection of less residual AP in the tube compared to the citronellol-based experiment further supports the conclusion that the myrtenol-based NCIL promotes a more complete reaction (Fig. S8). These results highlight the importance of the choice of chiral selector in achieving optimal enantioselectivity and reaction efficiency in electrochemical synthesis. Both NCILs demonstrated efficacy in the enantioselective electrosynthesis of *R*-PE, but the myrtenol-based NCIL showed enhanced reactivity, suggesting that differences in the structure of the chiral selector can significantly impact the outcome of the reaction.

## Mechanistic origin of enantioselectivity

To provide direct evidence for the “favorable diastereomeric interactions” responsible for the observed enantioselectivity, a computational investigation was performed using Density Functional Theory (DFT).^23–25^ The transition states for the reduction of AP to both *R*- and *S*-PE were modelled within a cluster containing the substrate and one ion pair of the respective NCIL. Calculations were performed at the B3LYP-D3/6-311+G(d,p) level of theory with an IEFPCM continuum solvation model (full details are provided in the SI).

The results, summarized in Table S1 and Scheme S1, are in excellent agreement with the experimental findings. For both NCILs, the calculated Gibbs free energy of activation (Δ*G*^‡^) for the pathway leading to *R*-PE (TS-R) is significantly lower than for the pathway to *S*-PE (TS-S), confirming the observed stereochemical preference (Scheme S1).

Critically, the energy difference between the diastereomeric transition states (ΔΔ*G*^‡^) is substantially larger for the myrtenol-based NCIL (−2.1 kcal mol^−1^) than for the citronellol-based one (−1.5 kcal mol^−1^). This directly translates to a higher predicted enantiomeric excess (93% *vs.* 82%) and provides a clear rationale for the superior performance of the myrtenol-NCIL observed experimentally.

Analysis of the optimized transition state geometries reveals the molecular origin of this difference. In the favored TS-R for the myrtenol system, the AP is locked into a highly organized arrangement within a rigid chiral pocket formed by the bicyclic pinane structure of the myrtenol group. This conformation is stabilized by hydrogen bonding and forces the electrochemical reduction to occur on one specific face of the ketone. The alternative pathway to the *S*-enantiomer is strongly disfavored due to a significant steric clash between the substrate's phenyl group and the myrtenol backbone. In contrast, the greater conformational flexibility of the citronellol chain creates a less defined chiral environment, resulting in a smaller energetic penalty for the formation of the *S*-enantiomer and thus, a lower overall enantioselectivity. These computational results provide robust, molecular-level evidence that the enantioselectivity is governed by the structural rigidity and steric demands of the NCIL's chiral component.

## Dynamic stereocontrol by tuning the electric field

**Fig. 3:**
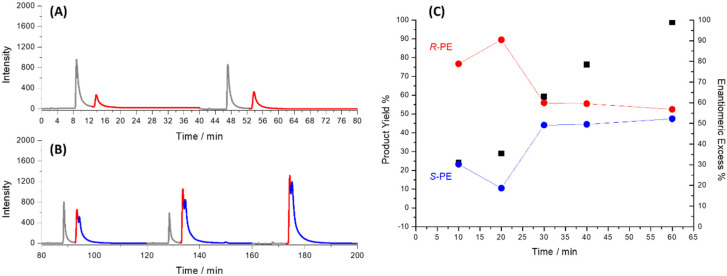
Chromatograms of the fractions collected after (A) 10 and 20 minutes and (B) 30, 40 and 60 minutes from the *δ*^+^ extremity of the Ppy tube with citronellol-based NCIL by applying a constant electric field of 2.8 V cm^−1^. The red and blue colors stand for *R*- and *S*-PE, respectively, while the grey peaks are related to AP. In (C) the corresponding enantiomeric excess (in red or blue) and apparent product yield (in black) as a function of the time of electrolysis are reported.

To explore the possibility of modulating the product selectivity, BE was performed with an increased electric field (2.8 V cm^−1^) using the citronellol-based NCIL (Fig. 3). The rationale behind increasing the electric field was to potentially overcome the chiral bias imposed by the NCIL configuration and promote the formation of both enantiomers. HPLC analysis of the collected fractions confirmed the formation of both *R*-PE and *S*-PE, indicating that increasing the electric field can indeed influence the stereochemical outcome of the reaction. The observation that the *R*-enantiomer was initially produced in greater quantities (Fig. 3A), even at the higher electric field, suggests that the chiral influence of the NCIL is not completely negated. The NCIL still appears to favor the formation of the *R*-enantiomer, although to a lesser extent than at lower electric fields. This result implies that while the electric field can be used to influence the enantiomeric ratio, the chiral environment provided by the NCIL continues to play a role in the stereochemical outcome. Quantitative analysis of Fig. 3 reveals that the ee and product yield evolve distinctly over time under a higher electric field (2.8 V cm^−1^).

After 10 minutes, the reaction shows a high ee (∼80%) in favor of the *R*-enantiomer, indicating the dominant influence of the NCIL at early stages. However, as electrolysis proceeds, the *S*-enantiomer begins to form in increasing amounts. This shift is not merely due to a loss of selectivity, but rather to the activation of a second, competing process leading to *S*-PE formation, which becomes accessible only at higher electric fields, as also suggested by CV enantioselectivity measurements. Although the *R*-pathway remains favored, the stronger electric field enables the system to overcome the chiral bias and initiate the *S*-enantiomer formation as well. As a result, by around 30 minutes the ee drops and stabilizes around 50%, reflecting the contribution of both enantiomers. At the same time, the apparent product yield increases continuously, exceeding 90% after 60 minutes, with minimal residual of AP (Fig. S9). This indicates that the stronger field not only modulates enantioselectivity but also enhances overall conversion. These findings demonstrate that BE under tunable electric fields offer a powerful strategy to steer both reaction rate and stereochemical outcome.

This experiment demonstrates the ability to modulate the product selectivity in BE by adjusting the applied electric field, offering a degree of control over the stereochemical outcome of the reaction.

## Substrate scope investigation

To evaluate the generality and potential synthetic utility of our wireless electrochemical microreactor, we extended our investigation to a series of prochiral ketones (Table S5). These substrates were strategically selected to probe the system's tolerance to varied steric and electronic demands, using the superior myrtenol-based NCIL under the optimized conditions (2.0 V cm^−1^). The results, summarized in Table S5 and Fig. S10, demonstrate the versatility of the method. The reduction of propiophenone, a close homologue of acetophenone, proceeded smoothly to afford 1-phenyl-1-propanol in high yield (91%) and enantioselectivity (90% ee). This indicates that a modest increase in steric bulk adjacent to the carbonyl group is well-tolerated. The influence of electronic effects was investigated using substituted acetophenones. The electron-deficient 4′-chloroacetophenone underwent rapid reduction, reaching near-quantitative conversion (>99%) while maintaining excellent enantioselectivity (95% ee). This is consistent with the increased electrophilicity of the carbonyl carbon accelerating the reaction. Conversely, the electron-donating group in 4′-methoxyacetophenone slowed the reaction, resulting in a lower conversion (85%), though the enantioselectivity remained high at 92% ee. These results show a predictable electronic influence on the reaction rate without compromising the chiral induction. Crucially, the protocol proved highly effective for heteroaromatic ketones, which are prevalent in pharmaceuticals. The reduction of 2-acetylthiophene yielded the corresponding alcohol with outstanding conversion (96%) and enantioselectivity (96% ee). To probe the steric limits of the chiral microenvironment, the bulky 2-acetylnaphthalene was also tested. As anticipated, the larger aromatic system resulted in a decrease in both conversion (75%) and enantioselectivity (80% ee), defining the boundaries of the method's effectiveness. Overall, this substrate scope study demonstrates that our wireless electrochemical method is a robust tool for the asymmetric reduction of a range of aromatic and heteroaromatic ketones, with predictable and rational outcomes based on substrate electronics and sterics.

## Green metrics section

To holistically evaluate the environmental performance of our proposed system, a green metrics analysis was conducted. The core enantioselective reduction of AP is an addition reaction, which achieves a theoretical Atom Economy of nearly 100%, indicating exceptional efficiency at the molecular level.

For a broader process perspective, we calculated the Process Mass Intensity (PMI) and introduced the Energy Intensity, which accounts for all mass and energy inputs used to generate the product. A detailed breakdown of the calculations, including an analysis of the feeder electrode current and associated side reactions, is provided in the SI. Based on the experimental data from a single microreactor run at 2.0 V cm^−1^ (Table S2), we determined a PMI of approximately 783 and an energy intensity of 38.7 kWh kg^−1^ of product.

While the energy and mass intensity values for this unoptimized, discovery-phase synthesis are notable, it is crucial to interpret them in context. Such values are not uncommon for laboratory-scale procedures where the primary goal is to establish proof-of-concept. A detailed analysis (Table S2) reveals that the primary contributors to the high PMI are the auxiliary bulk solvents used for the buffer and product extraction, which account for over 96% of the total input mass. This reframes the high PMI not as a failure of the core technology, but as a powerful diagnostic tool that clearly identifies the downstream workup protocol as the primary target for future process optimization.

Crucially, when benchmarked against conventional methods for acetophenone reduction (see SI, Table S3, for a detailed comparison), our electrochemical approach offers significant green advantages. It operates at ambient temperature and pressure, avoiding the safety hazards of high-pressure H_2_ gas used in catalytic hydrogenation. It also eliminates the use of expensive precious metal catalysts and avoids the poor atom economy and stoichiometric inorganic waste streams characteristic of metal hydride reductions. The primary byproducts from the electrical input are benign H_2_ and O_2_ from water electrolysis, representing a much cleaner waste profile (Table S4). This positions our tunable, wireless electrochemical system as a promising and sustainable platform for asymmetric synthesis.

## Conclusions

In conclusion, this work has successfully demonstrated that wireless bipolar electrosynthesis using NCILs as microreactors is a viable and innovative platform for highly enantioselective synthesis. The unique combination of electromechanical pumping within a chiral ionic liquid medium, coupled with the ability to modulate stereoselectivity by tuning the external electric field, introduces a powerful and green strategy for asymmetric transformations. While this study serves as a successful proof-of-concept, transitioning this method toward practical significance requires addressing several key challenges. Future work should focus on optimizing reaction parameters to improve conversion efficiency, as residual starting material was observed, potentially due to mass transport limitations imposed by the viscosity of the NCILs. Furthermore, while the core reaction is highly mass-efficient, the overall Process Mass Intensity (PMI) is high due to the solvents used in the lab-scale workup protocol; developing scalable, low-solvent extraction and purification strategies is therefore essential for enhancing the process's green credentials.^26^ Finally, although preliminary tests showed good reusability, rigorous long-term stability and lifetime studies of the Ppy microreactors are necessary to ensure their robustness for continuous or repeated use.

## Author contributions

Sara Grecchi: methodology, investigation, validation, data curation, writing – original draft, writing – review & editing. Andrea Mezzetta: methodology, investigation, writing – review & editing. Lorenzo Guazzelli: supervision, resources, writing – review & editing. Serena Arnaboldi: conceptualization, funding acquisition, supervision, resources, project administration, writing – review & editing.

## Conflicts of interest

There are no conflicts to declare.

## Supplementary Material

GC-027-D5GC04756K-s001

GC-027-D5GC04756K-s002

GC-027-D5GC04756K-s003

## Data Availability

The data supporting this article have been included as part of the supplementary information (SI). Supplementary information is available. The supplementary information includes detailed experimental procedures, spectroscopic data, and kinetic and optimization studies. See DOI: https://doi.org/10.1039/d5gc04756k.
