# Supplementary material for: Tunable stereoselectivity in a wireless electrochemical microreactor using natural chiral ionic liquids
Source: Green Chem. 2025 Oct 30;27(46):14769–75. doi: 10.1039/d5gc04756k (PMC12584937; doi:10.1039/d5gc04756k)
Supplement: GC-027-D5GC04756K-s001 [file GC-027-D5GC04756K-s001.pdf]

Supplementary Information (SI) for Green Chemistry.
This journal is © The Royal Society of Chemistry 2025

Supporting Information

Tunable Stereoselectivity in a Wireless Electrochemical Microreactor Using Natural Chiral Ionic Liquids

Sara Grecchi,[*a] Andrea Mezzetta,[b] Lorenzo Guazzelli [b] and Serena Arnaboldi[*a]

[a]Università degli Studi di Milano, Dipartimento di Chimica, Via Golgi 19, Milano 20133, Italia

[b]Università degli Studi di Pisa, Dipartimento di Farmacia, Via Bonanno 33, Pisa 56126, Italia

1. Materials and Methods (Video S2)

1) Design of the wireless enantioselective flow pumping reactor

For the polymerization of polypyrrole (Ppy), an aqueous solution containing 0.2 M pyrrole monomer and 0.25 M sodium dodecylbenzenesulfonate was prepared. The components were dissolved under magnetic stirring at a temperature not exceeding 40°C and then allowed to cool to room temperature.

Polymerization was performed in a three-electrode electrochemical cell with an Ag|AgCl reference electrode and a platinum counter electrode. The working electrode was a graphite rod (a common commercial pencil lead) about 3 cm long and 3 mm wide. It was coated with a thin layer of silver paint (EM-Tec AG15), which acted as a lubricant to facilitate the subsequent removal of the Ppy tube from the graphite electrode, used as a template.

The galvanostatic electropolymerization was carried out by applying a constant current of approximately 3 mA for 2 hours, ensuring the formation of sufficiently thick tube walls. During synthesis, the potential was maintained between 0.6 and 0.7 V to prevent polymer overoxidation.

After 2 hours, the graphite working electrode modified with Ppy, was removed from the electrochemical cell and washed with acetone to dissolve the silver paint and facilitate the detachment of the Ppy tube, which was then sonicated multiple times in acetone to remove any residual silver. Finally, the obtained tubes were cut to a uniform length of 1 cm and their edges were refined.

The schematic illustration of the fabrication steps is reported in Figure S1.

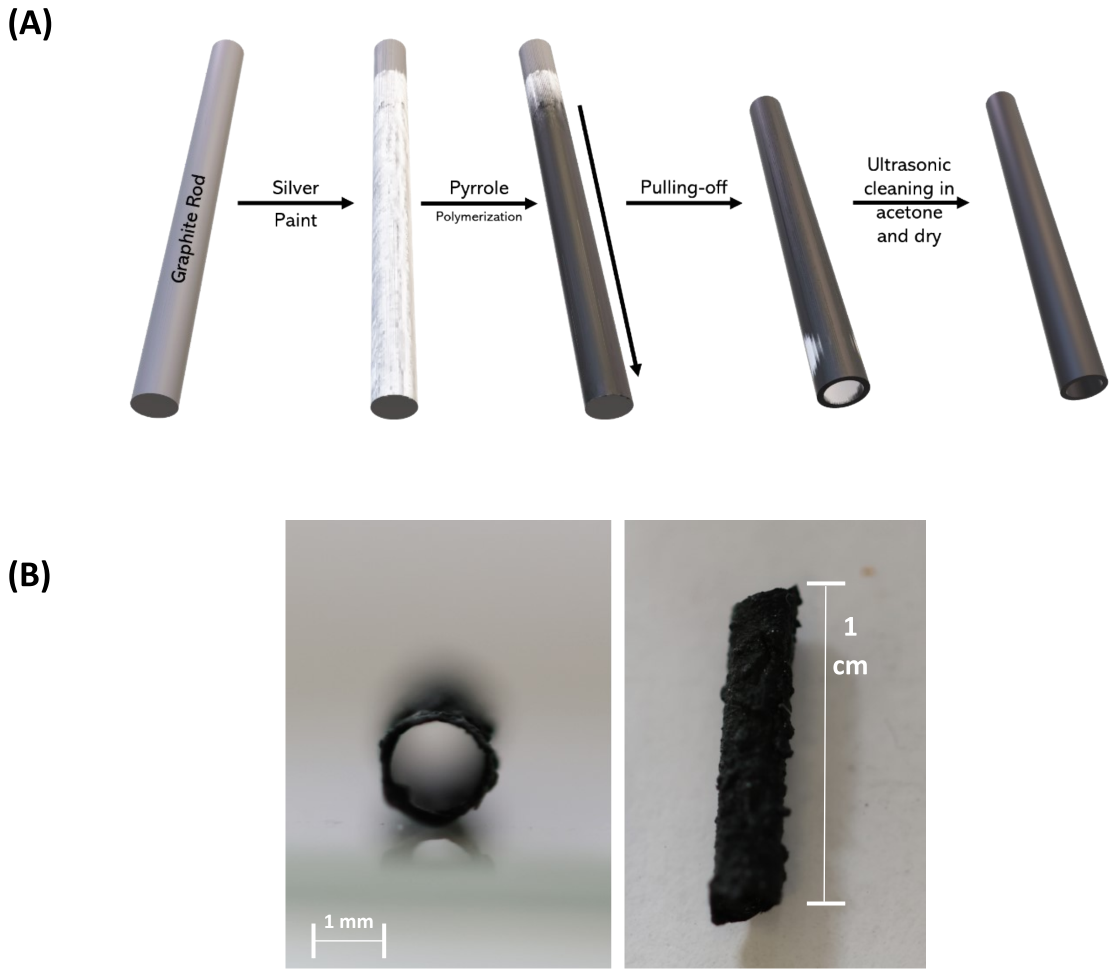

Figure S1. (A) Schematic illustration of the fabrication steps of the Ppy hollow tube. (B) Optical pictures of the hollow Ppy tube (front and top views).

2) Electrochemical characterization of NCILs

The two NCILs were initially characterized by cyclic voltammetry (CV) to investigate their electrochemical properties and identify possible redox reactions involving the molecular structure of the compound. These CV experiments were carried out using a PalmSens potentiostat, dissolving the citronellol- or myrtenol-based NCILs (0.00075 M) in an acetonitrile (ACN, Aldrich, HPLC grade) solution with 0.1 M tetrabutylammonium hexafluorophosphate ($TBAPF_6$, Fluka ≤ 98%) as the supporting electrolyte. The three-electrode minicell consisted of a glassy carbon (GC) working electrode, a platinum wire counter electrode, and an Ag|AgCl reference electrode placed in a double bridge filled with the working medium and ending with a porous frit to prevent KCl leakage into the working solution.

3) Potential window and enantioselection tests towards the enantiomers of PE

The subsequent CV experiments, including the determination of the potential window of the two NCILs and the enantioselectivity measurements, were conducted using screen-printed electrodes (SPEs), which are disposable electrodes printed on small-sized substrates. These electrodes incorporate a complete electrochemical cell, consisting of a working electrode and a counter electrode (both graphite in this case) and a "pseudo-reference" electrode ($Ag|Ag^+$). The electrodes were using 48-electrode sheets and a DEK 245 screen-printing machine (Weymouth, UK) on a flexible polyester film (Autostat HT5) obtained from Autotype Italia (Milan, Italy). A graphite-based ink (Electrodag 421) from Acheson (Milan, Italy) was used to print the working and counter electrodes, while a silver/silver chloride ink (Acheson Electrodag 4038 SS) was used to print the reference electrode. The diameter of the SPE working electrode is 0.4 cm, resulting in an apparent geometric area of 0.126 cm^2. The measurements for determining the potential window of the two NCILs based on myrtenol and citronellol were carried out by depositing a 6 µL drop of sample onto the three electrodes of the SPE at a scan rate of 50 mV/s, deaerating with N_2 to prevent interference from the oxygen reduction voltammetric signal and to preserve the molecules from potential degradation.

Conversely, the enantioselectivity tests were performed (also on SPEs) by utilizing these chiral ionic liquids based on myrtenol or citronellol as a chiral bulk medium in which the enantiomers of 1-phenylethanol (*R*- and *S*-PE, 2 mM) were dissolved. After sonicating the solutions, a 6 µL drop was deposited onto the three electrodes of the SPE, and the scan was performed in the reduction direction at 50 mV/s, with the entire setup continuously deaerated with N_2.

4) Wireless enantioselective electrosynthesis of PE

A 1 cm (l) Ppy tube was attached to an inert support at the center of a bipolar electrochemical cell, with two graphite feeder electrodes positioned at its ends, 5 cm (L) apart, and connected to a power supply.

The cell was then filled with a 0.2 M lithium perchlorate ($LiClO_4$) buffer solution at pH 4, serving as the supporting electrolyte, while 10 µL of the prochiral precursor (i.e., acetophenone, AP) was added to 100 µL of the chiral ionic liquid based on citronellol (or myrtenol). A constant electric field of 2 V/cm, calculated by dividing the applied voltage from the power supply by the 5 cm distance between the feeder electrodes, was applied for approximately one hour, and 30 µL of the AP solution dissolved in the chiral ionic liquid was injected via micropipette at the anodic end (δ^-) of the Ppy tube. Throughout the experiment, several fractions were collected at the cathodic end (δ^+) of the tube, extracted using 3 mL of heptane, and subsequently analyzed by chiral HPLC.

After the experiment, the tube was sonicated in heptane to detect any remaining traces of compounds inside through chiral HPLC. Finally, by increasing the applied electric field to 2.8 V/cm while maintaining a reaction time of one hour, the racemic mixture of PE was synthesized. This experiment was conducted using citronellol-based chiral ionic liquid. As before, the AP + chiral ionic liquid solution was injected at δ^-, while the collected fractions at δ^+ were analyzed by chiral HPLC.

5) Chiral High-Performance Liquid Chromatography (HPLC)

Chiral HPLC analyses were carried out with a HPLC equipment (Agilent 1260 Infinity II) coupled with a Daicel CHIRALPAK IG-3 column in isocratic reverse phase conditions. The HPLC analyses of the collected fractions at both different reaction times and applied electric fields were carried out by injecting 10 µL of each solution in the chiral column by using hexane/2-propanol (Hex/IPA) 99:1 as the mobile phase and a 0.5 mL/min flow. The photodiode array (PDA) detector was operating at a wavelength of 210 nm. Moreover, AP (i.e. the prochiral precursor), as well as the two antipodes of PE were also tested separately as such, under the same conditions in order to compare the enantioseparation results with the starting material and to correctly assign the chromatographic peaks to the corresponding enantiomers.

6) Morphological and elemental analysis of the soft Ppy tubes

The Ppy tubes were characterized by scanning electron microscopy (SEM) and energy dispersive X-ray (EDX) spectroscopy, using a Vega3 Tescan 20.0 kV microscope, in order to *i*) investigate and determine their morphology, as well as the wall thickness and the inner/outer diameters and *ii*) identify the elemental composition of the Ppy tubes for evaluating the effectiveness of the experimental procedure in removing silver residues following the use of silver paint as a lubricant.

7) DFT Calculations

Computational studies were performed using Density Functional Theory (DFT) to investigate the mechanism and enantioselectivity of the electrochemical reduction of acetophenone (AP). The transition states for the formation of both R-phenylethanol (*R*-PE) and S-phenylethanol (*S*-PE) were modeled.

The calculations were performed on a model system consisting of a cluster containing the substrate (AP) and one ion pair of the respective natural chiral ionic liquid (NCIL). All calculations were carried out at the B3LYP-D3/6-311+G(d,p) level of theory. The effect of the solvent was included using the Integral Equation Formalism variant of the Polarizable Continuum Model (IEFPCM).

The Gibbs free energies of activation ($\Delta G^{\ddagger}$) for the transition states leading to the R- and S-products (TS-R and TS-S) were calculated to predict the energetic favorability and the resulting enantiomeric excess.

8) Substrate Scope Investigation: General Procedure and Characterization Data

Materials Propiophenone (≥97.0%), 4'-Chloroacetophenone (97%), 4'-Methoxyacetophenone (99%), 2-Acetylthiophene (≥98%), and 2-Acetylnaphthalene (≥99%) were purchased from Sigma-Aldrich and Thermo Scientific and used as received without further purification.

General Procedure for Wireless Enantioselective Electrosynthesis The enantioselective reduction of the new prochiral ketones was carried out following the same procedure established for acetophenone. In a typical experiment, a 1 cm Ppy tube was placed in the bipolar electrochemical cell filled with 0.2 M lithium perchlorate ($LiClO_4$) buffer solution (pH 4). A solution of the respective prochiral ketone (10 µL) in the myrtenol-based NCIL (100 µL) was prepared. 30 µL of this solution was injected at the anodic end (δ^-) of the Ppy tube. A constant electric field of 2.0 V/cm was applied for 60 minutes. Fractions were collected from the cathodic end (δ^+) at 10-minute intervals, extracted with heptane (3 mL), and analyzed by chiral HPLC to determine the conversion and enantiomeric excess.

Chiral High-Performance Liquid Chromatography (HPLC) Analysis Analyses were performed on an Agilent 1260 Infinity II system equipped with a Daicel CHIRALPAK IG-3 column (250 mm x 4.6 mm, 3 µm). The mobile phase was a mixture of hexane/2-propanol (Hex/IPA) 99:1 (v/v) at a flow rate of 0.5 mL/min. The injection volume was 10 µL, and detection was performed at 210 nm. The absolute configuration of the major enantiomer was determined by comparison with known standards or literature data.

The retention times (tR) for each substrate and its corresponding alcohol enantiomers under these conditions are listed below:

- **Acetophenone (AP) / 1-Phenylethanol (PE)**
    - t R (AP): 5.1 min
    - t R (S-PE): 7.2 min
    - t R (R-PE): 7.9 min
- **Propiophenone / 1-Phenyl-1-propanol**
    - t R (Propiophenone): 4.5 min
    - t R (S)-1-Phenyl-1-propanol: 6.0 min
    - t R (R)-1-Phenyl-1-propanol: 6.5 min
- **4'-Chloroacetophenone / 1-(4-Chlorophenyl)ethanol**
    - t R (4'-Chloroacetophenone): 5.0 min
    - t R (S)-1-(4-Chlorophenyl)ethanol: 7.2 min
    - t R (R)-1-(4-Chlorophenyl)ethanol: 7.8 min
- **4'-Methoxyacetophenone / 1-(4-Methoxyphenyl)ethanol**
    - t R (4'-Methoxyacetophenone): 5.5 min
    - t R (S)-1-(4-Methoxyphenyl)ethanol: 8.0 min
    - t R (R)-1-(4-Methoxyphenyl)ethanol: 8.8 min
- **2-Acetylthiophene / 1-(Thiophen-2-yl)ethanol**
    - t R (2-Acetylthiophene): 4.0 min
    - t R (S)-1-(Thiophen-2-yl)ethanol: 5.5 min
    - t R (R)-1-(Thiophen-2-yl)ethanol: 6.2 min
- **2-Acetylnaphthalene / 1-(Naphthalen-2-yl)ethanol**
    - t R (2-Acetylnaphthalene): 7.0 min
    - t R (S)-1-(Naphthalen-2-yl)ethanol: 9.5 min
    - t R (R)-1-(Naphthalen-2-yl)ethanol: 10.5 min

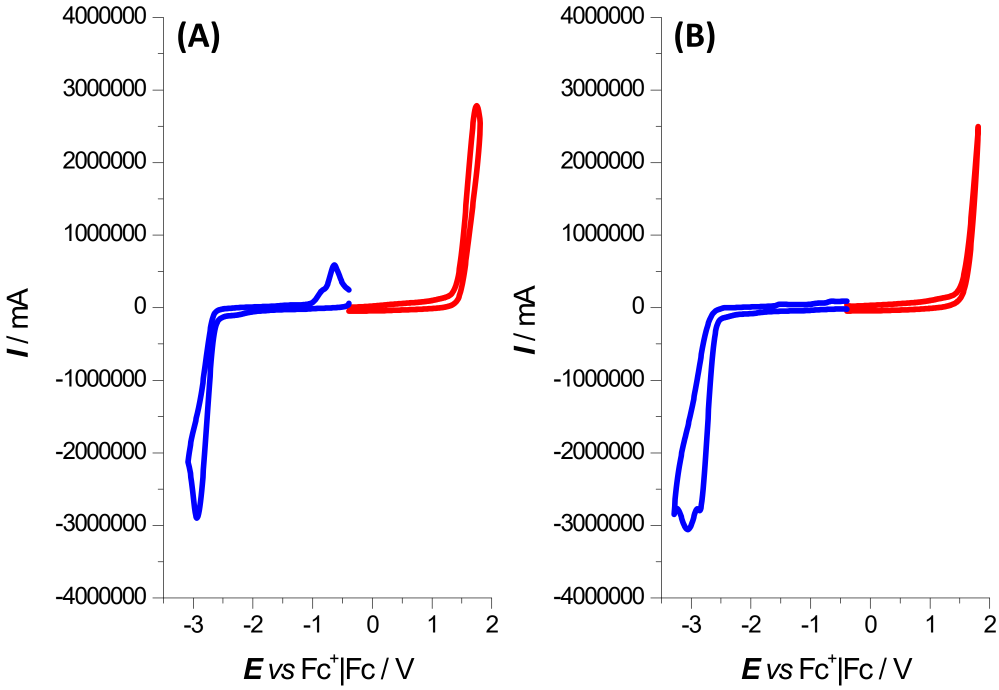

Figure S2. CV features of the two NCILs, (A) citronellol-based and (B) myrtenol-based, recorded at 0.2 V/s scan rate, in 0.00075 mol/dm^3 solutions in ACN+$TBAPF_6$ 0.1 mol/dm^3. Potentials have been normalized *vs* the formal potential of the intersolvental ferricenium|ferrocene (Fc^+|Fc) reference redox couple, recorded in the same condition. The experiments were carried out in a three-electrode cell by using GC as the working electrode, Ag|AgCl and Pt wire as the reference and counter electrodes, respectively.

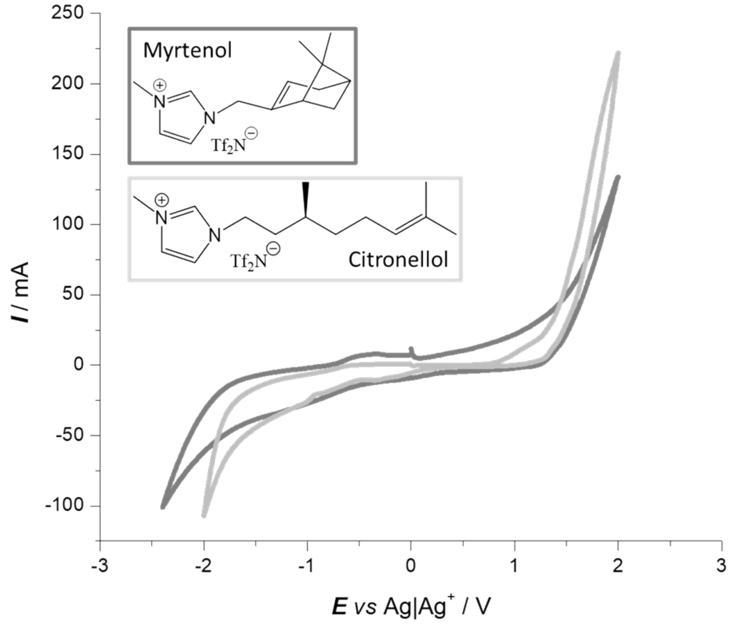

Figure S3. CV potential windows (at 0.05 V/s scan rate) of bulk NCILs based on citronellol (light grey) and myrtenol (dark grey), as thin films spread on screen printed electrode cells with graphite working and counter electrodes and Ag|Ag^+ pseudoreference electrode.

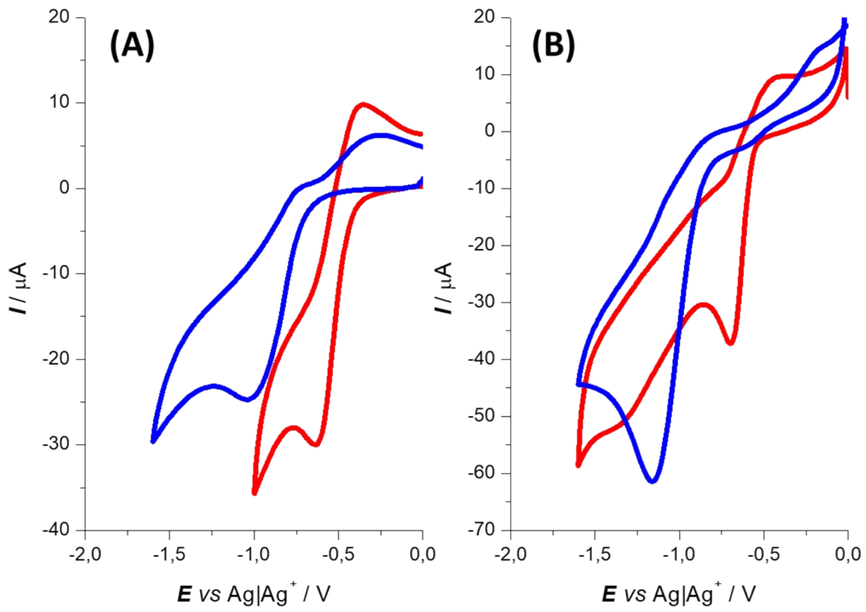

Figure S4. Enantioselection test with 2 mM *R*- and *S*-PE (red and blue lines, respectively) on a graphite SPEs (50 mV/s scan rate) in NCILs based on citronellol (A) or myrtenol (B) solutions.

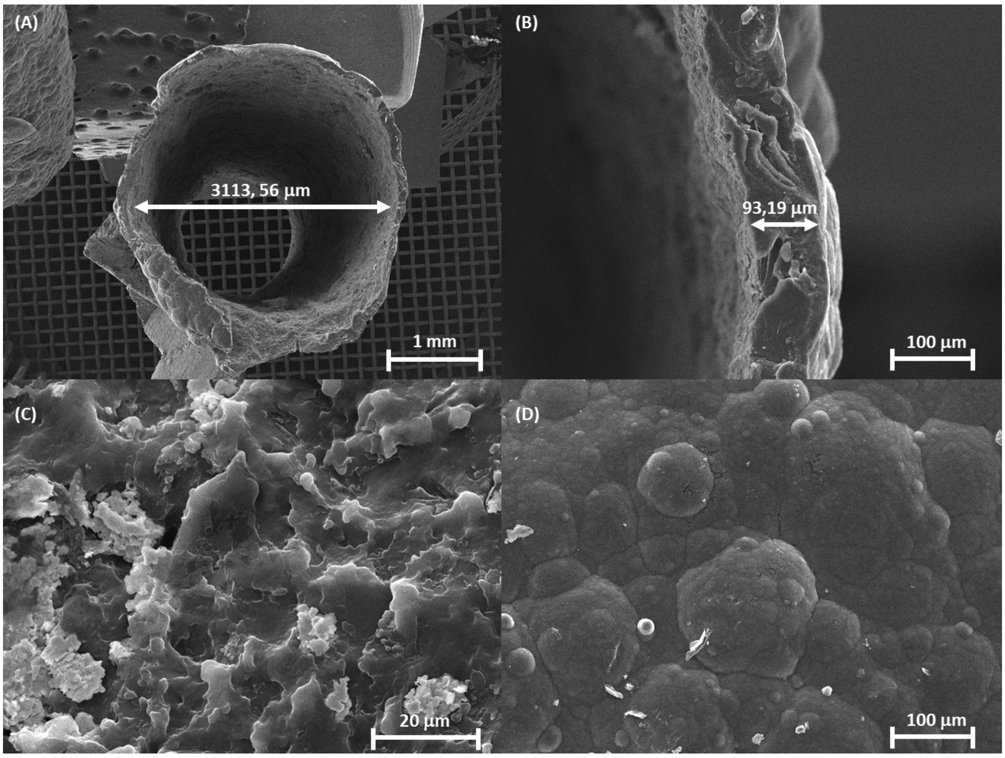

Figure S5. SEM micrographs of the (A) front view of one extremity of the Ppy tube, with the relative dimensions of the inner diameter value (scale bar 1 mm), (B) wall thickness (scale bar 100 μm), (C) internal (scale bar 20 μm) and (D) external part (scale bar 100 μm) of the Ppy tube.

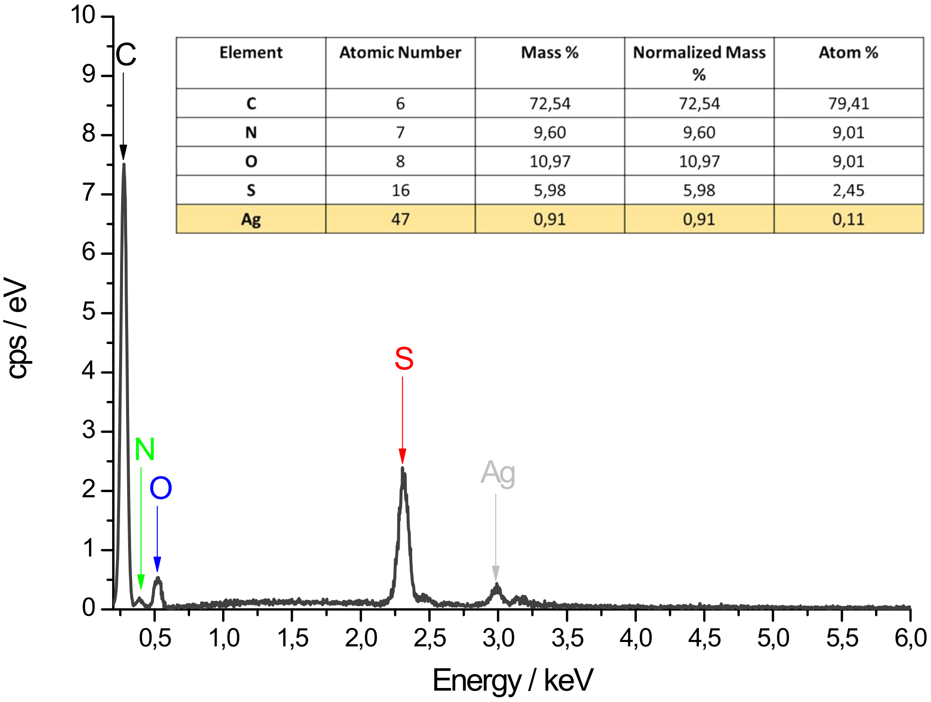

Element	Atomic Number	Mass %	Normalized Mass %	Atom %
C	6	72,54	72,54	79,41
N	7	9,60	9,60	9,01
O	8	10,97	10,97	9,01
S	16	5,98	5,98	2,45
Ag	47	0,91	0,91	0,11

Figure S6. EDX signals around the emission peak of carbon, nitrogen, oxygen, sulphur and silver of the Ppy tube.

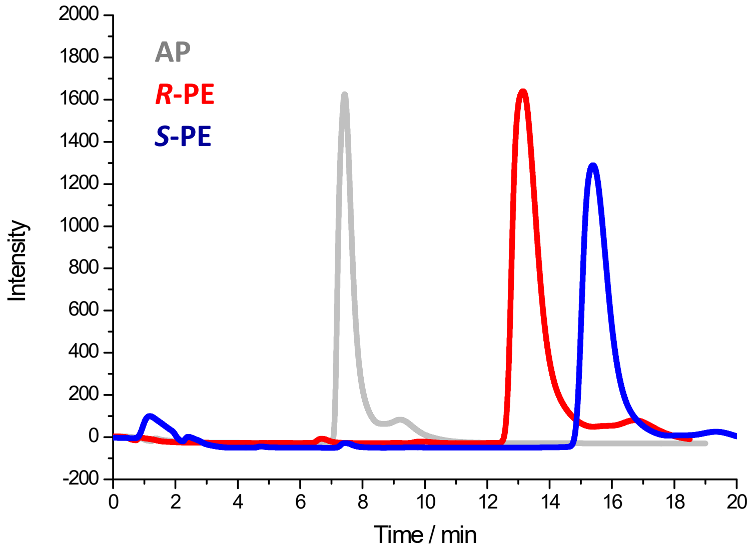

Figure S7. Chromatograms related to the direct HPLC analyses of pristine AP (in gray), *R*- and *S*-PE (in red and blue, respectively).

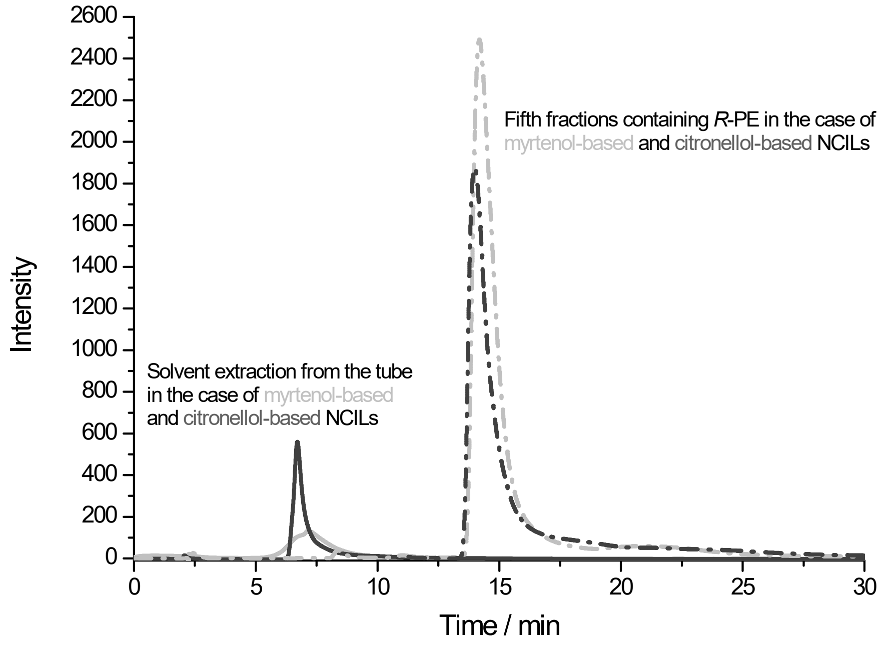

Figure S8. Chromatograms of the heptane solution (solid line) used to wash the tubes after the BE experiments (by applying a constant electric field of 2.0 V/cm for 60 minutes), carried out with myrtenol- or citronellol-based NCILs (light or dark grey, respectively). The dotted lines (light or dark grey, corresponding to myrtenol- or citronellol-based NCILs, respectively) represent the chromatograms of the last fractions, containing *R*-PE, collected during the electrolysis.

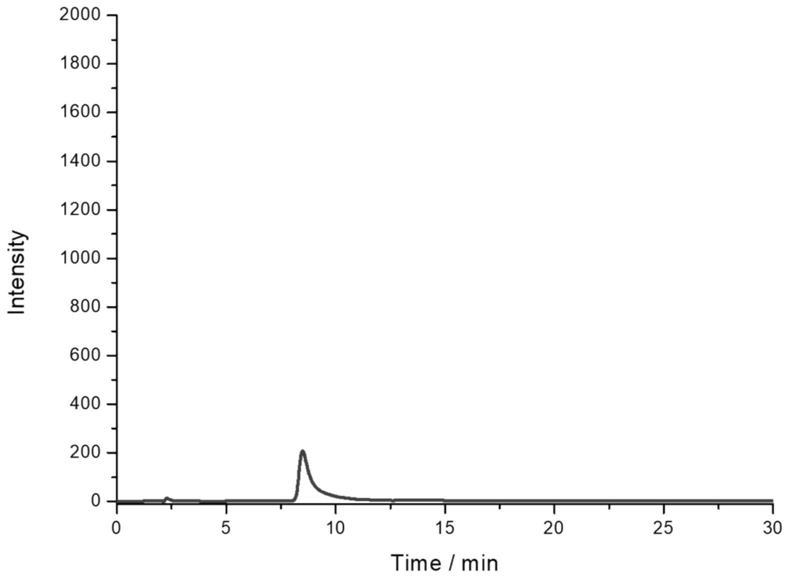

Figure S9. Chromatogram of the heptane solution used to wash the tube after the BE experiments (by applying a constant electric field of 2.8 V/cm for 60 minutes), carried out with citronellol-based NCIL.

Video S1. Electrochemical pumping of a NCIL droplet through the Ppy tube under a constant applied electric field.

Video S2. Experimental steps from the asymmetric reactor preparation to HPLC analysis

Chiral Ionic Liquid	Transition State	ΔG‡ (kcal/mol)	ΔΔG‡ (kcal/mol)	Predicted ee (%)
**Myrtenol-NCIL**	**TS-R** (to *R*-PE)	**21.3**	**-2.1**	**93% (*R*)**
	**TS-S** (to *S*-PE)	**23.4**		
**Citronellol-NCIL**	**TS-R** (to *R*-PE)	**22.5**	**-1.5**	**82% (*R*)**
	**TS-S** (to *S*-PE)	**24.0**		

Table S1. Calculated Activation Energies and Predicted Enantioselectivity.

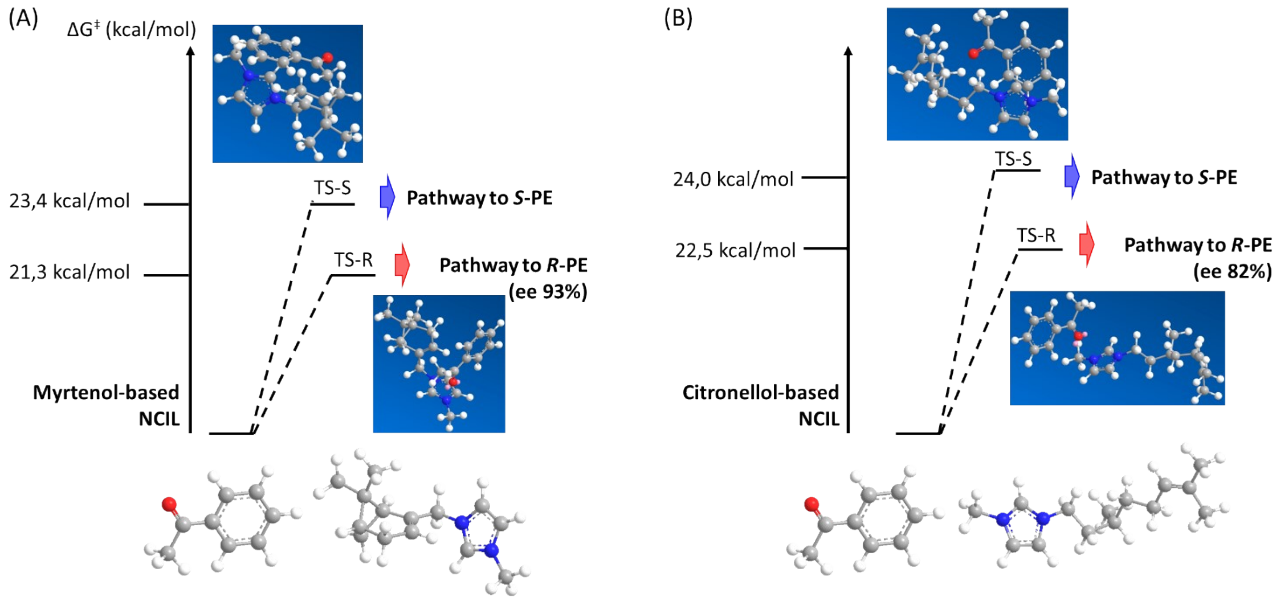

Scheme S1. Reaction pathways for the enantioselective reduction of AP in (A) myrtenol-based and (B) citronellol-based NCILs, showing the distinct transition states and associated ΔG‡ values leading to preferential formation of *R*-PE.

Enantiomeric excess

The enantiomeric excess (%ee) was calculated by using equation (1):

$$Enantiomeric\ Excess\ (\%\ ee) = \frac{(R)PE\ - (S)PE}{(R)PE + (S)PE} \times 100 \quad (1)$$

where (*R*)-PE and (*S*)-PE represent the corresponding integrated HPLC peak areas.

Product yield value

The product yield (% PY) of each compound present in the sample was evaluated by the ratio between the integrated HPLC peak area of *R*-enantiomer and the sum of the integrated HPLC peak areas of the precursor and of the *R*-enantiomer (Equation 2):

$$\%\ PY\ (R)\text{-}enantiomer = \frac{Area\ of\ R - enantiomer}{Total\ Area} \times 100 \quad (2)$$

In this work we have used this equation to calculate the PY, because AP and PE have almost the same molar extinction coefficient (ε_{AP} = 12 mM^{-1} cm^{-1} and ε_{PE} = 11.9 $mM^{-1}cm^{-1}$).

2. Analysis of the Feeder Current and Energy Consumption

The foundation of a comprehensive green assessment for an electrochemical process is the quantification of its energy demand. The "wireless" nature of the bipolar electrode (BPE) refers to the absence of a direct physical connection to the power source; however, the system as a whole requires an external electric field to function. This field is established by passing a current through a bulk supporting electrolyte via two feeder electrodes.

The magnitude of the ionic current flowing through the electrochemical cell is governed by Ohm's law, where the current is directly proportional to the specific conductivity, κ, of the electrolyte solution. The experimental procedure specifies a 0.2 M aqueous solution of lithium perchlorate at pH 4 as the supporting electrolyte.

While a direct literature value for this specific concentration and pH is not immediately available within the provided documentation, a robust estimate can be derived using the principle of molar conductivity, κ, which relates the specific conductivity to the molar concentration, Λ, via the equation $\Lambda=\kappa/c$. Molar conductivity is a measure of an electrolyte's efficiency in conducting current per mole of ions. For strong, fully dissociated 1:1 electrolytes in aqueous solution, Λ values are of a similar order of magnitude and exhibit a predictable, slight decrease with increasing concentration due to inter-ionic interactions.

Therefore, data for a chemically similar, strong 1:1 electrolyte, such as lithium chloride (LiCl), can serve as a reasonable proxy to estimate the molar conductivity of $LiClO_4$. Published data [1] indicate that a 0.09523 M (~0.1 M) aqueous solution of LiCl at 25 °C has a specific conductivity of κ=9.019 mS·cm^{-1}.

Acknowledging the slight decrease in molar conductivity with increasing concentration, a conservative value of Λ≈90 S·cm^{2}·mol^{-1} is a justifiable estimate for a 0.2 M solution. This assumption is further supported by the fact that the ionic mobilities of the chloride and perchlorate anions are comparable. Using this estimated molar conductivity, the specific conductivity of the 0.2 M $LiClO_4$ solution can be calculated.

This value, equivalent to 18 mS·cm^{-1} or 1.8 S·m^{-1}, is consistent with the conductivity ranges reported for various aqueous and non-aqueous electrolytes of similar concentrations, confirming it as a reliable basis for subsequent calculations.

The experimental setup employs two graphite feeder electrodes positioned at a distance (L) of 5 cm apart within the electrolyte solution. Experiments were conducted under two different applied electric fields (ε): 2.0 V/cm and 2.8 V/cm. The total voltage (V) applied across these electrodes is the product of the electric field and the separation distance (V= ε x L).

For the 2.0 V/cm condition: V=2.0 V/cm×5 cm=10.0 V

For the 2.8 V/cm condition: V=2.8 V/cm×5 cm=14.0 V

The total current (I) flowing through the cell is the product of the current density (J) and the cross-sectional area of the current path (A), where the current density is itself the product of the specific conductivity and the electric field (J=κ x ε). Therefore, I= κ × ε × A. The cell with a simple prismatic cell geometry was filled with 5 mL of the buffer solution, where this 5 mL (5 cm^{3}) volume is distributed over the 5 cm length between the electrodes, the effective cross-sectional area of the current path can be estimated as A= 1cm^{2}.

Using this geometry and the estimated conductivity, the total current can be calculated for both experimental conditions:

For the 2.0 V/cm condition: 36 mA

For the 2.8 V/cm condition: 50.4 mA

The electrical power (P) consumed by the system is the product of the applied voltage and the resulting current (P=V×I).

For the 2.0 V/cm condition: 0.36 W

For the 2.8 V/cm condition: 0.71 W

These calculations reveal a crucial aspect of the system's operation. The primary energy driver is not the microscopic reaction within the Ppy tube, but the bulk electrolysis of the entire 5 mL of supporting electrolyte solution required to establish the potential gradient. This reframes the "wireless" nature of the BPE: while the Ppy tube is galvanically isolated, the system as a whole possesses a significant and quantifiable energy demand. This understanding immediately identifies a key target for future process optimization: miniaturization of the entire electrochemical cell, not just the microreactor, would proportionally reduce the electrolyte volume and thus the total current and power required to maintain the same electric field.

Furthermore, reporting the electric field in V/cm is fundamentally more informative than reporting total voltage, as it describes the intrinsic driving force experienced by the BPE, independent of cell geometry. The analysis based on the electric field allows for greater scalability and generalization of the results. It also suggests that process efficiency could be improved by redesigning the cell to decrease the electrode gap (L) while maintaining the optimal electric field (ε), thereby lowering the required voltage (V) and power (P) consumption.

The total energy consumed during a standard 60-minute (3600 s) experiment is a critical green metric. This is calculated as the product of the power and the reaction time (Energy = P x t).

For the 2.0 V/cm condition: 1296 J

For the 2.8 V/cm condition: 2542 J

To facilitate comparison with standard energy benchmarks, these values can be converted to kilowatt-hours (kWh), where 1 kWh = 3.6×10^6 J.

For the 2.0 V/cm condition: 3.6×10^{-4} kWh

For the 2.8 V/cm condition: 7.1×10^{-4} kWh

3.Side Reactions and Byproduct Formation

The applied cell voltages of 10.0 V and 14.0 V far exceed the thermodynamic potential required for the electrolysis of water. To identify the dominant reactions, all plausible redox processes at the anode and cathode must be considered.

Possible Cathodic (Reduction) Reactions:

1. **Reduction of water:** $2H_2O(l)+2e^- \rightarrow H_2(g)+2OH^-(aq)$. The standard potential is -0.83 V at pH 14. At pH 4, the potential is given by the Nernst equation:

   $E=E^\circ-(0.059/2)\log([H_2][OH^-]^2)=0-(0.059/2)\log((1)/(10^{-4})^2)=-0.236$ V vs. SHE.

2. **Reduction of lithium ions:** $Li^+(aq)+e^- \rightarrow Li(s)$, with a standard potential of $E^\circ=-3.04$ V vs. SHE. This reaction is highly unfavorable compared to water reduction.

3. **Reduction of perchlorate ions:** $ClO4^-(aq)+H_2O(l)+2e^- \rightarrow ClO_3^-(aq)+2OH^-(aq)$, with a standard potential of E°=+0.17 V vs. SHE in basic solution. While thermodynamically plausible, the reduction of the perchlorate anion is known to be subject to an extremely high kinetic barrier. The chlorine atom in the +7 oxidation state is sterically shielded by four oxygen atoms, making electron transfer exceptionally slow on most electrode surfaces, including graphite. Perchlorate is widely used as a "non-interfering" or "inert" supporting electrolyte in aqueous electrochemistry precisely for this reason.

Possible Anodic (Oxidation) Reactions:

1. **Oxidation of water:** $2H_2O(l) \rightarrow O_2(g)+4H^+(aq)+4e^-$. The standard potential is +1.23 V vs. SHE at pH 0. At pH 4, the potential is E=1.23−0.059×4=+0.994 V vs. SHE.

2. **Oxidation of chloride ions (from impurities):** $2Cl^-(aq) \rightarrow Cl_2(g)+2e^-$, with a standard potential of E°=+1.36 V vs. SHE. This is less favorable than water oxidation and would only occur if significant chloride impurities were present.

3. **Oxidation of the graphite electrode:** $C(s)+2H_2O(l) \rightarrow CO_2(g)+4H^+(aq)+4e^-$. While possible, this process typically occurs at higher potentials or over long-term operation and is not considered the primary reaction under these conditions with stable graphite electrodes.

Based on this thermodynamic and kinetic assessment, the reduction of water to hydrogen gas at the cathode and the oxidation of water to oxygen gas at the anode are the overwhelmingly dominant electrochemical reactions occurring at the feeder electrodes. The total cell potential required for water electrolysis is $E_{cell}=E_{anode}-E_{cathode}$=(+0.994 V)−(−0.236 V)=1.23 V. The applied voltages are more than sufficient to overcome this thermodynamic barrier as well as any associated overpotentials at the graphite electrodes. Therefore, these byproducts can be confidently identified as gaseous hydrogen and oxygen avoiding the generation of toxic organic, halogenated, or solid salt byproducts that are characteristic of many conventional chemical reductions. From a waste-stream perspective, this makes the process inherently cleaner.

With the current calculated above, Faraday's laws of electrolysis can be used to determine the precise amount of hydrogen and oxygen generated during a 60-minute (3600 s) experiment. The total charge (Q) passed through the cell is given by Q = I x t.

For the 2.0 V/cm condition (I= 0.036 A): Q=129.6 C

For the 2.8 V/cm condition (I = 0.0504 A): Q=181.4 C

The number of moles of product formed is calculated using the formula: Moles = Q/(n×F), where n is the number of electrons transferred per mole of product and F is the Faraday constant (96485 C/mol).

Hydrogen Generation (Cathode):

At 2.0 V/cm: Moles H_2= 6.72×10^{-4} mol

At 2.8 V/cm: Moles H_2= 9.40×10^{-4} mol

Oxygen Generation (Anode):

At 2.0 V/cm: Moles O_2= 3.36×10^{-4}mol

At 2.8 V/cm: Moles O_2= 4.70×10^{-4}mol

These molar quantities can be converted to mass and volume at standard temperature and pressure (STP: 0 °C and 1 atm, molar volume = 22.4 L/mol).

Mass of Byproducts: (Molar mass of H_2 ≈ 2.02 g/mol; O_2 ≈ 32.00 g/mol)

At 2.0 V/cm: Mass H_2 = 1.36 mg

Mass O_2 = 10.75 mg

Total Mass = 12.11 mg

At 2.8 V/cm: Mass H_2 = 1.90 mg

Mass O_2 = 15.04 mg

Total Mass = 16.94 mg

Volume of Byproducts (STP):

At 2.0 V/cm: Volume H_2 = 15 mL

Volume O_2 = 7.5 mL

At 2.8 V/cm: Volume H_2 = 21 mL

Volume O_2 = 10.5 mL

This quantification confirms that a measurable, but chemically simple, set of byproducts is generated. The total mass of water consumed in this process is 12.11 mg and 16.94 mg for the two conditions, respectively, which is a negligible fraction of the 5000 mg of water in the buffer solution.

4. Comparative Green Metrics Evaluation

**Material**	**Role**	**Amount (per run)**	**Density (g/mL, approx.)**	**Mass (mg)**	**Input Category**
Acetophenone (AP)	Reactant	10 µL	1.03	10.3	Raw Material
NCIL (Citronellol-based)	Chiral Medium	100 µL	1.1 (est.)	110	Auxiliary
Lithium Perchlorate	Electrolyte	0.2 M in 5 mL	-	106.4	Auxiliary
Water (for buffer)	Solvent	5 mL	1.0	5000	Solvent
Heptane	Extraction Solvent	3 mL	0.684	2052	Workup Solvent
**Total Input Mass**				**7278.7 mg**	

R-Phenylethanol (PE)	Product	90% yield	-	9.3 mg	Product
Water Consumed (Electrolysis)	Byproduct Precursor			12.1 mg	Waste
Hydrogen Gas Generated	Byproduct			1.36 mg	Waste
Oxygen Gas Generated	Byproduct			10.75 mg	Waste
**PMI**	**(Total Input / Product)**			**~783**	
**E-Factor**	**(PMI - 1)**			**~782**	

Table S2. Green Chemistry Metrics Analysis for the Enantioselective Synthesis of *R*-PE

The Energy Intensity, defined as the energy consumed per gram of product, is an essential metric. Using the total energy consumption and the product mass (9.3×10^{-6} kg), this can be calculated for the high-selectivity (2.0 V/cm) experiment:

Energy Intensity = 38.7 kWh/kg

This value provides a quantitative measure of the process's energy efficiency. While this figure may appear high, its significance can only be understood through comparison with the energy demands of alternative technologies. It is also critical to consider the source of this energy. If the electricity is generated from renewable sources (e.g., solar, wind), the carbon footprint of the process is minimal. If it is derived from fossil fuels, the environmental impact is substantial.

Process Mass Intensity (PMI) and E-Factor

PMI = total mass of inputs (raw materials, solvents, reagents, process aids)/mass of final product (3)

E-Factor = total mass of waste/mass of product (where E-Factor = PMI - 1) (4)

Atom Economy (AE)

The electrosynthesis of PE is a reduction reaction, which is a type of addition reaction.

The overall transformation is:

C_8H_8O (Acetophenone) + $2H^+$ + $2e^-$ → $C_8H_{10}O$ (Phenylethanol)

In essence, this is the addition of two hydrogen atoms across the carbonyl double bond.

The theoretical atom economy is therefore calculated as:

AE = (Molecular Weight of Product) / (Sum of Molecular Weights of Reactants) x 100% (5)

AE = (MW of $C_8H_{10}O$) / (MW of C_8H_8O + MW of H_2) x 100% AE = (122.16 g/mol) / (120.15 g/mol + 2.02 g/mol) x 100% ≈ 100% (6)

The proposed electrochemical synthesis of chiral phenylethanol can be benchmarked against three major conventional approaches: catalytic hydrogenation, stoichiometric reduction with metal hydrides, and biocatalysis.

Metric	**Bipolar Electrosynthesis**	**Catalytic Hydrogenation**	**Stoichiometric Reduction ($NaBH_4$)**	**Biocatalysis**
**Reaction Conditions**	Ambient temperature and pressure	High pressure (up to 200 atm) and high temperature (up to 250 °C) [2]	Ambient temperature and pressure [3]	Mild, physiological temperature and pressure [4,5]
**Primary Energy Source**	Electricity (grid or renewable)	Heat and compression (typically fossil fuels)	Embodied energy in the chemical reductant	Embodied energy in nutrients/glucose for cell growth
**Reagent Toxicity & Hazards**	Low (water, $LiClO_4$). Gaseous H_2/O_2 produced in situ.	High (flammable/explosive H_2 gas, pyrophoric catalysts, organic solvents) [6]	Moderate ($NaBH_4$ is water-reactive, flammable solid; H_2 evolution during quench)	Low (enzymes, buffers, glucose are generally non-toxic)
**Catalyst**	None (reagent-free at the BPE)	Precious metals (Pd, Pt, Ru) or base metals (Ni, Co) [2,7,8]	None (stoichiometric reagent)	Whole cells or isolated enzymes
**Atom Economy (Theoretical)**	~100% (addition reaction)	~100% (addition reaction)	Very poor (large mass of Na, B, O atoms are waste)	N/A (complex biological system)
**E-Factor (Typical Range)**	High (~782) due to lab-scale workup; potentially low if optimized.	Moderate to high, dominated by solvent and catalyst processing.	Very high, dominated by stoichiometric borate waste	Can be extremely high due to dilute aqueous media and complex purification [5]

**Key Waste Streams**	Aqueous salt solution, extraction solvent, benign H_2/O_2 gases.	Spent metal catalysts (heavy metal waste), organic solvent waste [9].	Aqueous borate salts ($NaBO_2$), solvent waste.	Large volumes of aqueous buffer, biomass, purification solvents.
**Selectivity**	High and tunable by electric field.	Can be low; risk of over-reduction to ethylbenzene or ring hydrogenation. [10]	High for carbonyl, but typically not enantioselective without chiral auxiliaries.	Extremely high (chemo-, regio-, and enantio-selectivity).

Table S3. Comparative Green Chemistry Assessment of Acetophenone Reduction Methodologies.

This comparative analysis reveals that the "greenness" of a process is not a monolithic property but a complex trade-off between multiple factors.

- **Catalytic hydrogenation**, while well-established, relies on hazardous high-pressure hydrogen, elevated temperatures, and often-toxic precious metal catalysts that pose disposal challenges.
- **Sodium borohydride reduction** is simple and effective but suffers from abysmal atom economy, generating a large stoichiometric stream of borate waste for every mole of product formed.
- **Biocatalysis** offers unparalleled selectivity under mild conditions but is often plagued by very low substrate concentrations, leading to massive E-Factors due to the large volumes of water and complex downstream processing required to isolate the product.

In this context, the electrochemical method presents a unique and compelling profile. Its primary drawback, a notable energy intensity in its unoptimized lab-scale form, is counterbalanced by significant advantages: operation at ambient temperature and pressure, the use of water as a solvent, the avoidance of hazardous chemical reductants or expensive metal catalysts, and the generation of a simple and benign waste stream (H_2/O_2 and a recyclable salt solution). The ability to tune enantioselectivity simply by adjusting an electrical parameter is a unique advantage not offered by the other methods.

5. Analysis of Feeder Current, Energy Consumption, and Associated Side Reactions

To provide a comprehensive assessment of the system's green credentials, the electrical current flowing between the feeder electrodes and the associated side reactions were analyzed. The ionic current is a function of the electrolyte's specific conductivity (κ) and the applied electric field (ε). The conductivity of the 0.2 M aqueous electrolyte (pH 4) was estimated to be based on literature [1] values for similar 1:1 electrolytes. Given the experimental geometry (5 cm electrode distance, estimated 1 cm^2 cross-sectional area), the total current, power, and energy consumption for a 60-minute experiment were calculated (Table S3).

Parameter	Symbol	Value (at 2.0 V/cm)	Value (at 2.8 V/cm)	Units
Applied Electric Field	ε	2.0	2.8	V/cm

Applied Voltage	V	10.0	14.0	V
**Calculated Current**	I	**36**	**50.4**	**mA**
**Calculated Power**	P	**0.36**	**0.706**	**W**
Total Energy per Run (60 min)	E_{total}	3.6×10^{-4}	7.1×10^{-4}	kWh

Table S4. Calculated Electrical Parameters for the Bipolar Electrochemical System.

A thermodynamic and kinetic analysis of all possible redox reactions in the aqueous system at pH 4 was conducted. Given the applied potentials (10 or 14 V) and the known high kinetic barrier for perchlorate reduction on non-catalytic surfaces, the overwhelmingly dominant side reactions are the electrolysis of water at the feeder electrodes:

- Cathode: $2H_2O_{(l)}+2e^- \rightarrow H_{2(g)}+2OH^-_{(aq)}$
- Anode: $2H_2O_{(l)} \rightarrow O_{2(g)}+4H^+_{(aq)}+4e^-$

Using Faraday's laws, the amount of H_2 and O_2 generated during a 60-minute run at 2.0 V/cm was calculated to be 1.36 mg (15 mL at STP) and 10.75 mg (7.5 mL at STP), respectively. These benign gaseous byproducts constitute the primary waste stream directly associated with the electrical input.

6. Stability and Operational Lifetime of the Polypyrrole (Ppy) Microreactor

The functioning of the Ppy microreactor is based on the reversible electrochemical process of doping and dedoping. In the oxidized (doped) state, the polymer backbone is positively charged and incorporates anions from the electrolyte to maintain charge neutrality, causing the material to swell. In the reduced (dedoped) state, electrons neutralize the backbone, and anions are expelled, causing it to contract. This reversible volumetric change drives the pumping effect utilized in our system.

The long-term stability of Ppy is primarily limited by an irreversible degradation process known as over-oxidation, which occurs if the polymer is exposed to excessively high anodic potentials. This process, caused by the attack of reactive species generated from water electrolysis, leads to the formation of carbonyl groups that disrupt the conjugation of the polymer backbone, resulting in a permanent loss of conductivity and electroactivity. For this reason, during the galvanostatic synthesis of our tubes, the potential was carefully monitored and maintained in the 0.6–0.7 V range to prevent the onset of such degradation.

The operational lifetime of Ppy-based devices, such as actuators, is typically quantified in thousands of operating cycles, with literature values ranging from 7,000 to over 30,000 cycles, depending on the operating conditions [11,12]. Our preliminary tests, in which the microreactors were reused with minimal loss of activity, are consistent with the robustness of the material when operated within its electrochemical stability window, confirming its suitability for the described application.

7. Substrate Scope Investigation

Substrate (Ketone)	NCIL	Product	Conversion (%)	ee (%)[b]	Config.[c]
Acetophenone[a]	Myrtenol	1-Phenylethanol	95	94	R
Acetophenone	Citronellol	1-Phenylethanol	92	85	R
Propiophenone[a]	Myrtenol	1-Phenyl-1-propanol	91	90	R
4'-Chloroacetophenone[a]	Myrtenol	1-(4-Chlorophenyl)ethanol	>99	95	R
4'-Methoxyacetophenone[a]	Myrtenol	1-(4-Methoxyphenyl)ethanol	85	92	R
2-Acetylthiophene[a]	Myrtenol	1-(Thiophen-2-yl)ethanol	96	96	R
2-Acetylnaphthalene[a]	Myrtenol	1-(Naphthalen-2-yl)ethanol	75	80	R
2-Acetylnaphthalene[d]	Citronellol	1-(Naphthalen-2-yl)ethanol	68	72	R

Table S5. Substrate scope for the enantioselective reduction of prochiral ketones. [a] Reactions performed with the myrtenol-based NCIL at 2.0 V/cm for 60 min. Apparent product yield determined by HPLC. [b] Enantiomeric excess determined by chiral HPLC. [c] Absolute configuration assigned by comparison with known standards. [d] Reaction performed with the citronellol-based NCIL for comparison.

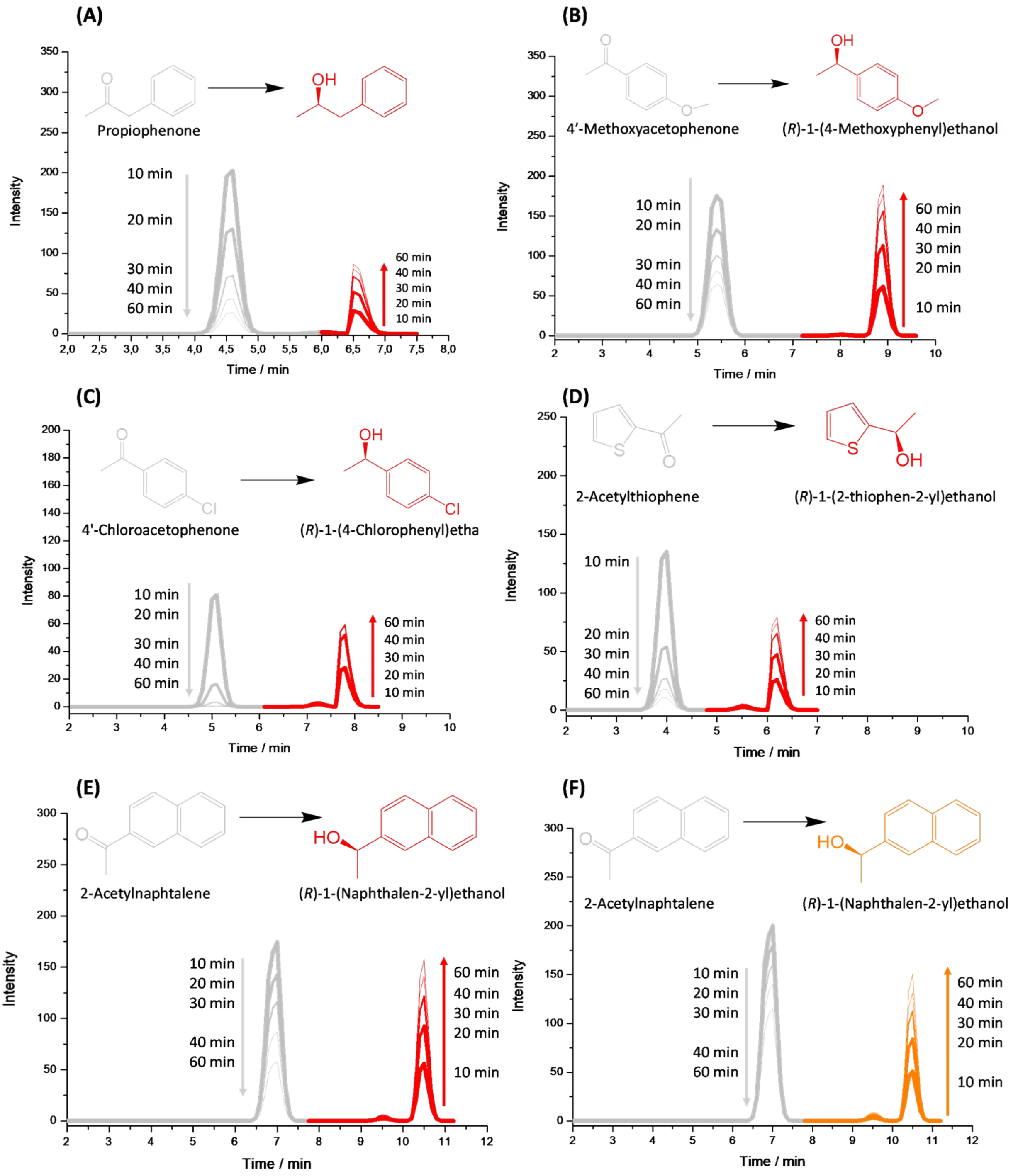

Figure S10. Overlaid HPLC chromatograms showing the reaction progress over 60 minutes for the reduction of: (A) Propiophenone, (B) 4'-Methoxyacetophenone, (C) 4'-Chloroacetophenone, (D) 2-Acetylthiophene, and (E) 2-Acetylnaphthalene using the myrtenol-based NCIL. (F) Reduction of 2-Acetylnaphthalene using the citronellol-based NCIL highlighting the impact of the chiral medium on reaction efficiency and selectivity. In each panel, grey peaks represent the starting prochiral ketone and colored peaks represent the (*R*)-alcohol product.

References

[1] CRC Handbook of Chemistry, and Physics, 70th Edition, Weast, R. C., Ed., CRC Press, Boca Raton, FL, 1989, p. D-221.

[2]10.1098/rsta.2020.0346

[3] Vol. 25 (5) May (2021) Res. J. Chem. Environ.

[4] 10.1515/acs-2015-0019

[5] 10.2174/0113852728240964231010103534

[6] 10.1021/acs.iecr.2c00003

[7] 10.1002/cssc.201901313

[8] **10.3390/catal14080545**

[9] 10.1080/01614940.2021.1926849

[10] J. Phys. Chem. C 2020, 124, 47, 25884–25891

[11] 10.3390/polym9090446

[12] 10.1117/12.715776
